# Proteome analysis enables separate clustering of normal breast, benign breast and breast cancer tissues

**DOI:** 10.1038/sj.bjc.6601008

**Published:** 2003-07-15

**Authors:** M V Dwek, A A Alaiya

**Affiliations:** 1 School of Biosciences, University of Westminster, 115 New Cavendish Street, London W1W 6UW, UK; 2 Unit of Cancer Proteomics, Department of Oncology and Pathology, Karolinska Institutet and Hospital, Stockholm, Sweden

**Keywords:** breast cancer, cluster analysis, proteome, two-dimensional electrophoresis

## Abstract

We have used proteomics with cluster analysis for the classification of breast tumour tissues. In our approach, we can distinguish between normal breast, benign breast and breast cancer tissues on the basis of the protein expression profiles. We propose an objective method for the classification of breast tumour specimens.

Proteomics is the main tool for understanding the vast amount of data generated from the human genome sequencing project. Proteomics is defined as the characterisation of all proteins encoded by the genome and allows identification of protein–protein interactions and disease-associated proteins.

Proteomic studies mainly use two-dimensional electrophoresis (2-DE) for protein separation and mass spectrometry for protein identification. Using 2-DE, a mixture of proteins is separated first on the basis of charge and second according to size. A typical 2-DE system can separate up to 3000 protein spots per experiment. Although this does not enable the entire proteome to be mapped in a single experiment, it is recognised as being unparalleled in terms of the data generated. In common with genetic microarray analysis, however, there is a need to comprehend the meaning of the vast amount of data that 2-DE experiments produce. Most proteome studies use univariate analysis to compare the levels of a protein spot in a cohort of diseased tissue samples compared with normal tissues. A newer approach is a refinement of multivariate analysis whereby expression levels of many proteins are compared simultaneously. Various methods, such as neural network and fuzzy logic have been proposed for this type of analysis (Alaiya *et al*, 2002).

The aim of our work was to identify proteins to differentiate between normal breast, benign breast and breast cancer tissue.

## MATERIALS AND METHODS

A mini 2-DE procedure was used to separate proteins from 32 normal and pathological breast specimens as well as axillary lymph node specimens removed following surgical excision.

The proteins were prepared and separated as described previously (Weekes *et al*, 1999). Isoelectric focusing was performed on nonlinear 3–10 IPG strips, 7 cm in size. The isoelectric focusing conditions were 10.5 kV h with the voltage set to 3000. The second-dimension gel electrophoresis was carried out in the Atto AE-6450 vertical electrophoresis system at 130 V for 2.5 h in tank buffer. The second-dimension gels were 8 cm in size and were run at 170 V for 1–1.5 h until the bromophenol blue marker had reached the bottom of the gel. After electrophoresis, the proteins in the gels were visualised by staining with silver nitrate using the PlusOne Protein Silver Staining Kit (Amersham Biosciences, Bucks, UK). The data from the gels were then imported into PDQuest software by laser-scanning densitometry (Bio-Rad, Hemel Hempstead, UK).

Quantitative data sets were analysed using the J-Express software (Alaiya *et al*, 2002). Hierarchical analysis allows samples that are highly similar to be merged in an agglomerative way, using the complete linkage clustering procedure. This grouping is presented in the form of dendrograms with trees and branches depicting the extent of similarities among the different groups in the samples. To generate distinct sample clusters, variables were selected using the Mann–Whitney statistical analysis between normal breast tissue and benign breast tissue (*P*<0.05). A similar analysis was conducted between groups of primary breast cancer specimens and axillary lymph node metastases.

## RESULTS

A total of 32 breast tissue samples were analysed by 2-DE; the clinical and histological characteristics of the samples are shown in Table 1

**Table 1:** Clinical and histopathological features of the specimens used in this study

**Sample no.**	**Tissue**	**Age of patient**	**Pathology**			
N1	Normal	57	Normal breast			
N2	Normal	56	Normal breast			
N3	Normal	24	Normal breast			
N4	Normal	27	Normal breast			
B1	Benign	42	Fibroadenoma			
B2	Benign	18	Fibroadenoma			
B3	Benign	28	Intraduct papillomatosis			
B4	Benign	68	Intraduct papillomatosis			
						
**Sample no.**	**Tissue**	**Age of patient**	**Pathology**	**Size**	**Grade**	**LN status**
C1	Cancer	52	Ductal carcinoma	19	G3	Not sampled
C2	Cancer	57	Ductal carcinoma	15	G3	0/6
C3	Cancer	77	Ductal carcinoma	20	G1	1/6
C4	Cancer	44	Ductal carcinoma	30	G2	1/17
C5	Cancer	70	Ductal carcinoma	20	Not stated	2/2
C6	Cancer	53	Ductal carcinoma	20	Not stated	3/8
C7	Cancer	51	Ductal carcinoma	100	G3	7/8
C8	Cancer	53	Ductal carcinoma	20	Not stated	Not sampled
C9	Cancer	56	Ductal carcinoma	40	Not stated	2/5
C10	Cancer	56	Ductal carcinoma	30	G2	6/8
C11	Cancer	71	Ductal carcinoma	25	Not stated	0/2
C12	Cancer	68	Advanced ductal cancer	150	Not sampled	Not sampled
C13	Cancer	79	Advanced ductal cancer	44	G3	4/4
C14	Cancer	78	Ductal carcinoma	28	G3	0/8
C15	Cancer	78				

LN=lymph node.

.

**Figure 1:**
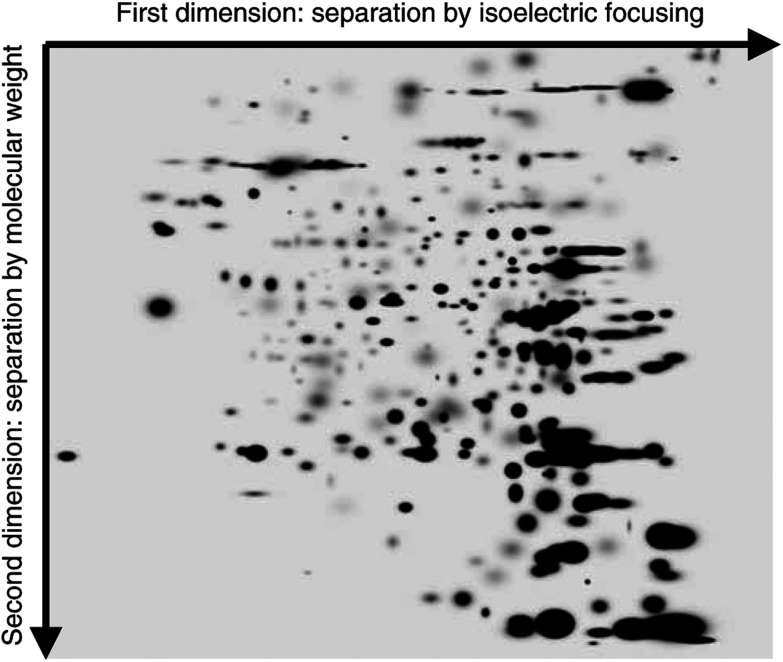
Reference gel showing proteins separated using two-dimensional electrophoresis. The proteins are separated first according to their isoelectric point and then separated according to size. The proteins were visualised using a silver stain.

The average number of resolved spots on the mini gels was 350 and the gel spots were matched between gels using a reference gel. The reference gel is shown in Figure 1

**Figure 2:**
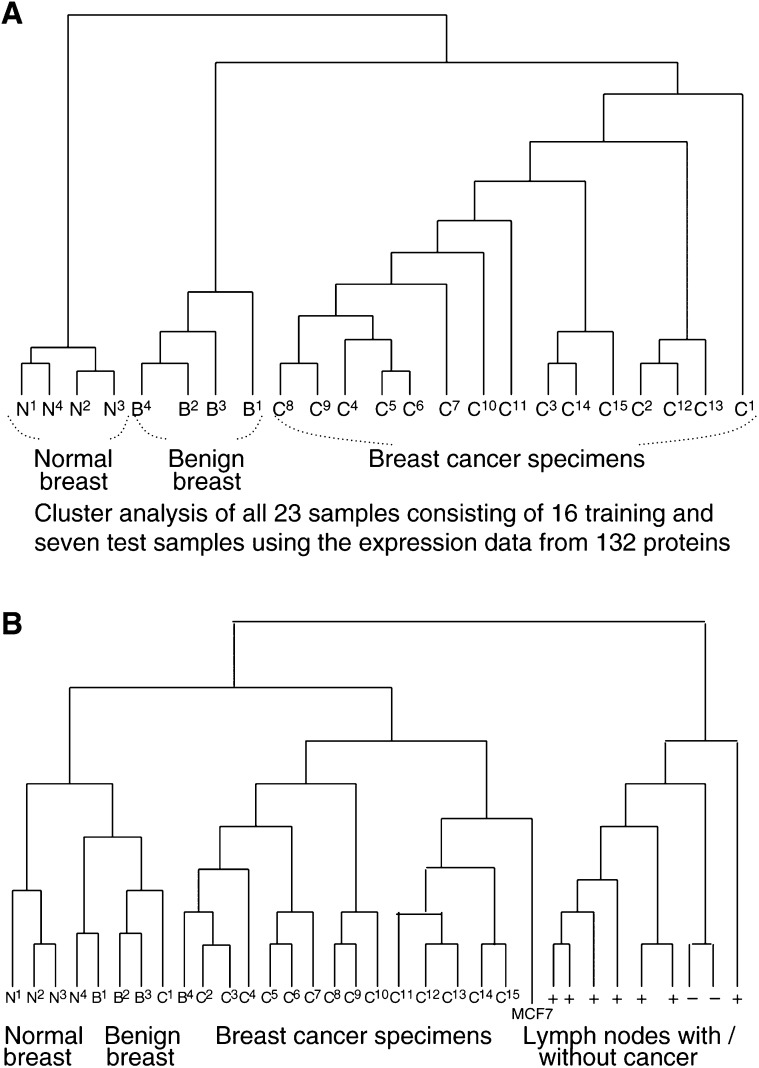
Cluster analysis of (**A**) 23 breast tumour samples using expression data from 132 protein spots and (**B**) all 31 breast tissue samples using expression data from 105 protein spots.

. In all, 16 breast tissue samples, consisting of three normal breast, three benign breast and 10 breast cancer, were initially analysed. A set of 132 spots differed significantly between normal breast tissue and benign breast tumours. This data set was used as the training set for the cluster analysis and all 16 samples were correctly classified (data not shown). We then tested if these markers could discriminate new cases by adding seven samples and the result was re-evaluated. All the 23 samples were correctly classified, as shown in Figure 2A

.

Next, we examined whether tumours with similar clinical outcomes could be classified. A set of nine breast tumours and MCF7, a breast cancer cell line, were added to the 23 samples. To improve the classification, a second variable selection was made. A total of 124 proteins were differentially expressed between primary breast cancer and the axillary node metastases (Mann–Whitney, *P*<0.05). This data set was then used for the hierarchical cluster analysis of all the 33 samples consisting of the five different sub groups. The dendogram produced from this analysis is presented in Figure 2B. The majority of the normal and benign tissues clustered together, while a branch of the dendrogram consisted exclusively of primary breast cancers, the third sub-branch consisted of a mixture of lymph nodes with or without metastatic breast cancer.

## DISCUSSION

This is the first time that 2-DE has been used with hierarchical cluster analysis for the classification of breast specimens, although a previous report using cells grown *in vitro* suggested that such analysis might be warranted (Harris *et al*, 2002).

We used mini-2-DE gels for the proteome analysis because the technology is rapid, simple and sensitive, making it especially applicable for routine diagnostic purposes. One disadvantage is that relatively few proteins could be resolved on mini gels. Clearly, the use of large size gels capable of resolving between 1000 and 2000 proteins in one experiment would have provided further data. It is tempting to speculate that the use of such gels would have enabled improved clustering, for example it may have separated the breast cancer cases according to the stage of their disease at presentation. The use of large gels, however, requires availability of larger amounts of starting material. We found that the small gel format was of sufficient resolution for cluster analysis to be performed, resulting in the separation of normal and benign breast tissue from breast cancer specimens on the basis of their protein-spot expression patterns.

A useful aspect of this analysis is that it allows the identification of the spots that contribute to the overall clustering. It seems unlikely, however, that a universal set of proteins will discriminate between different subgroups in a cohort of samples and it will be interesting to discover if training sets of selected variables remain a prerequisite for efficient sample classification. In this study, 33 spots were common to both dendrograms and when evaluated separately, these proteins enabled clustering of almost all the samples, therefore, these protein spots are obvious candidates for further study.

In summary, we have used mini-2-DE to separate proteins from breast tissue samples and found that hierarchical cluster analysis enabled discrimination between normal breast, benign breast tissue and breast cancer tissue specimens according to their protein expression profiles.
